# Knockdown of LncRNA *RHPN1-AS1* Inhibits Cell Migration, Invasion and Proliferation in Head and Neck Squamous Cell Carcinoma

**DOI:** 10.7150/jca.29029

**Published:** 2019-07-05

**Authors:** Xiaowen Qiu, Zhuo Lei, Zeyou Wang, Yuming Xu, Chang Liu, Panchun Li, Hanjiang Wu, Zhaojian Gong

**Affiliations:** 1 Department of Stomatology, Second Xiangya Hospital, Central South University, Changsha 410011, Hunan, China;; 2 Department of Stomatology, Haikou people's Hospital, Haikou 570208, Hainan, China;; 3 Xiangya Stomatological Hospital & School of Stomatology, Central South University, Changsha 410008, Hunan, China;; 4 Department of Laboratory Medicine, Second Xiangya Hospital, Central South University, Changsha 410011, Hunan, China.

**Keywords:** Long non-coding RNA, *RHPN1-AS1*, HNSCC, EMT

## Abstract

Previous studies have revealed that long non-coding RNAs (lncRNAs) are involved in head and neck squamous cell carcinoma (HNSCC) progression. However, the detailed roles of lncRNA *RHPN1-AS1* remain to be elucidated. In this study, by analyzing online RNA-Seq data, we found that *RHPN1-AS1* was upregulated in HNSCC tissues and that its expression level was associated with neoplasm histologic grade. High expression of *RHPN1-AS1* was also confirmed in HNSCC tissues. Knockdown of *RHPN1-AS1* inhibited tumor cell migration, invasion and proliferation in HNSCC. Furthermore, inhibition of *RHPN1-AS1* suppressed the expression of epithelial-mesenchymal transition (EMT)-related genes (*β-Catenin*, *Claudin-1* and *Vimentin*) in HNSCC cells. Collectively, our results suggest that *RHPN1-AS1*, acting as an oncogene, may be a potential diagnostic and therapeutic target in HNSCC.

## Introduction

Squamous cell carcinoma is one of the most aggressive cancers worldwide and has a poor prognosis despite recent advances in therapeutics 1. Head and neck squamous cell carcinoma (HNSCC) accounts for up to 90% of all head and neck cancers derived from the oral cavity, oropharynx and hypopharynx and is the sixth leading cancer worldwide, with an estimated 600,000 new cases annually 2, 3. Patients usually have no obvious signs in the early stage of the disease, resulting in delayed diagnosis. Although surgery, radiation therapy and adjuvant chemotherapy are used to treat HNSCC according to National Comprehensive Cancer Network (NCCN) guidelines, tumor control and patient survival are poor 4. Therefore, identification of early biomarkers and elucidation of the tumor process of HNSCC will be helpful for improving survival while maintaining patient quality of life.

Long non-coding RNAs (lncRNAs) are newly discovered non-coding RNAs with more than 200 nucleotides and no protein coding ability 5. In recent years, accumulating evidence has revealed that lncRNAs function as tumor suppressors or promoters in carcinogenesis and cancer progression among different cancer types 6-10. The lncRNA *AFAP1-AS1* is upregulated and acts as an oncogene in a variety of cancers 11. The lncRNAs *CCAT1* and* CCAT2*, located in the 8q24.21 locus, were highly overexpressed in colorectal cancer and were significantly associated with recurrence-free survival (RFS) and overall survival (OS), which serve as important prognostic biomarkers in colorectal cancer 12. The lncRNA *DANCR* increased cancer stem cell function by upregulating AXL/PI3K-Akt via competitive binding to miR-33a-5p in osteosarcoma 13. We analyzed the TANRIC online HNSCC data 14 and found that *RHPN1-AS1* was significantly upregulated in HNSCC tissues, suggesting that *RHPN1-AS1* may function as a tumor promoter in this cancer. However, the relationship between *RHPN1-AS1* and HNSCC has not been previously reported.

In this article, we identified the *RHPN1-AS1* expression level and detailed functions in HNSCC. Our study might provide a promising new strategy for RNA-based diagnosis and therapies in HNSCC.

## Materials and methods

### Data mining and analysis

The online data of *RHPN1-AS1* expression level and clinical characteristics were obtained from the TANRIC platform (http://ibl.mdanderson.org/tanric/_design/basic/index.html). The search strategy was described in a previous report 14. The correlation analysis between *RHPN1-AS1* and the *β-Catenin*, *Claudin-1* and *Vimentin* expression levels in HNSCC was analyzed by using the Gene Expression Profilling Interactive Analysis (GEPIA) database 15 (http://gepia.cancer-pku.cn/index.html).

### Tissue specimens and cell lines

HNSCC tissues and adjacent normal tissues (located > 1 cm away from the tumor) were obtained from 26 patients who underwent HNSCC resection at Second Xiangya Hospital, Central South University. These tissues were immediately frozen in liquid nitrogen and stored at -80°C until analysis. Informed consent was obtained from each patient, and the study was approved by the Ethics Committee of Second Xiangya Hospital, Central South University. The human HNSCC cell lines Cal-27 and Tca8113 were maintained in DMEM and RPMI-1640 medium supplemented with 10% fetal bovine serum and antibiotics (100 units/ml penicillin and 100 µg/ml streptomycin). Cells were incubated at 37°C in a humidified incubator containing 5% CO_2_.

### Short interfering RNA (siRNA) and cell transfection

Knockdown of *RHPN1-AS1* was performed using siRNAs provided by RiboBio (Guangzhou, China). The siRNA sequences were as follows: *RHPN1-AS1*-siRNA1-sense: CCGAAUCUCUUUACUUCCAdTdT, *RHPN1-AS1*-siRNA1-antisense: UGGAAGUAAAGAGAUUCGGdTdT; *RHPN1-AS1*-siRNA2-sense: CUCAAACUUUGAGGGUCAUdTdT, *RHPN1-AS1*-siRNA2-antisense: AUGACCCUCAAAGUUUGAGdTdT. The cells were cultured overnight and transfected with siRNAs using Lipofectamine RNAiMAX Reagent (Invitrogen; Thermo Fisher Scientific, Inc.) in accordance with the manufacturer's instructions. For validation of the transfection efficiency, lncRNA expression was examined by quantitative real-time PCR (qPCR).

### QPCR

Total RNA from cells and tissues was extracted with TRIzol reagent (Invitrogen; Thermo Fisher Scientific, Inc.) according to the manufacturer's protocol. Reverse-transcribed *RHPN1-AS1* cDNA was obtained using a Revert Aid™ First Strand cDNA synthesis kit (Fermentas; Thermo Fisher Scientific, Inc.). QPCR analyses were performed according to a previous study 11. Samples were compared using the relative CT method, where the relative expression of *RHPN1-AS1* was normalized to that of *β-Actin*. The primer sequences were as follows: *RHPN1-AS1* forward, 5'- GCTCCTGGTCATCAAGTTCCTCT-3' and reverse, 5'- GCACAGGCACCAGAATGATCC-3'; *β-Actin* forward, 5'-TCACCAACTGGGACGACATG-3' and reverse, 5'-GTCACCGGAGTCCATCACGAT-3'.

### Cell proliferation assay

The 3-(4,5-dimethylthiazol-2-yl)-2,5-diphenyltetrazolium bromide (MTT) assay was used to measure cell proliferation. After transfection, 5 × 10^3^ cells/well cells were seeded in 96-well plates and cultured for 1, 2, 3 and 4 days, and then, the supernatant was removed, and MTT (5 mg/ml, 20 μl) was added to each well at 37˚C. After 4 h, 100 μl dimethyl sulfoxide (DMSO) was added to each well. The optical density (OD) value was determined at 490 nm by a microplate reader and used to construct a growth curve to assess cell proliferation.

### Τranswell assays

Transwell migration and invasion assays were performed to measure the migration and invasive capacity of the transfected cells. In brief, cells were plated on 8-µm pore size membranes with or without Matrigel (2 × 10^4^ cells/well in serum-free medium), which were in turn placed in the top chamber of 24-well transwell plates. The bottom chamber contained 800 µl chemotactic factor. After 24 h, cells on the upper surface were removed, while cells attached to the membranes were fixed in 4% paraformaldehyde for 20 min and stained with hematoxylin. The results of the transwell assay were imaged, and the number of invasive cells was evaluated by manual counting.

### Flow cytometry analysis

For analysis of the cell cycle, cells were collected after transfection for 24 h and fixed in precooled 75% ethanol overnight at 4°C. After the cells were washed with 1 × phosphate buffer solution (PBS) (1,000 rpm, 5 min), they were resuspended in a solution of 800 µl of 1 × PBS containing 1% bovine serum albumin (BSA). After addition of 100 µl of PI dye (3.8 × 10^-2^ M sodium citrate, pH 7.0) and 100 µl of RNase A (10 mg/ml), cells were incubated at 37°C for 30 min in the dark. Cell cycles were analyzed by flow cytometry. For detection of apoptosis, cells were digested with 0.25% EDTA-free trypsin and collected by centrifugation at 1,000 rpm for 5 min. After cells were washed with 1 × PBS, 2 × 10^5^ cells were suspended in 500 µl of binding buffer. Then, 5 µl of annexin V-FITC was added, followed by the addition of 5 µl of propidium iodide, and the samples were incubated at room temperature for 10 min in the dark room. Apoptotic cells were sorted by flow cytometry.

### Western blot assay

Cells were collected 48 h after transfection with *RHPN1-AS1* siRNA or control siRNA. Then, the cells were washed twice with precooled PBS. After RIPA cleaved the cells, we used a BCA kit to measure the protein concentration. Denatured proteins (30 μg) were electrophoresed on 10% sodium dodecyl sulfate-polyacrylamide gel (SDS-PAGE). After completion of electrophoresis, the protein was transferred onto polyvinylidene fluoride (PVDF) membranes. After the membranes were blocked with 5% milk for 1 h, they were incubated with primary anti-β-catenin, claudin-1, and vimentin antibodies at 4°C overnight. The next day, membranes were washed with PBS and incubated with secondary antibodies at 37°C for 1 h. Intensity of protein expression was detected by ECL chemiluminescence.

### Statistical analysis

All experiments were repeated at least three times. Statistical analyses were carried out using SPSS 19.0 software (SPSS, Chicago, IL, USA). The results were assessed by Student's t-test. All statistical tests were two-sided, and a *P*-value of < 0.05 was considered statistically significant.

## Results

### LncRNA *RHPN1-AS1* was upregulated in HNSCC tissues

**Figure 1:**
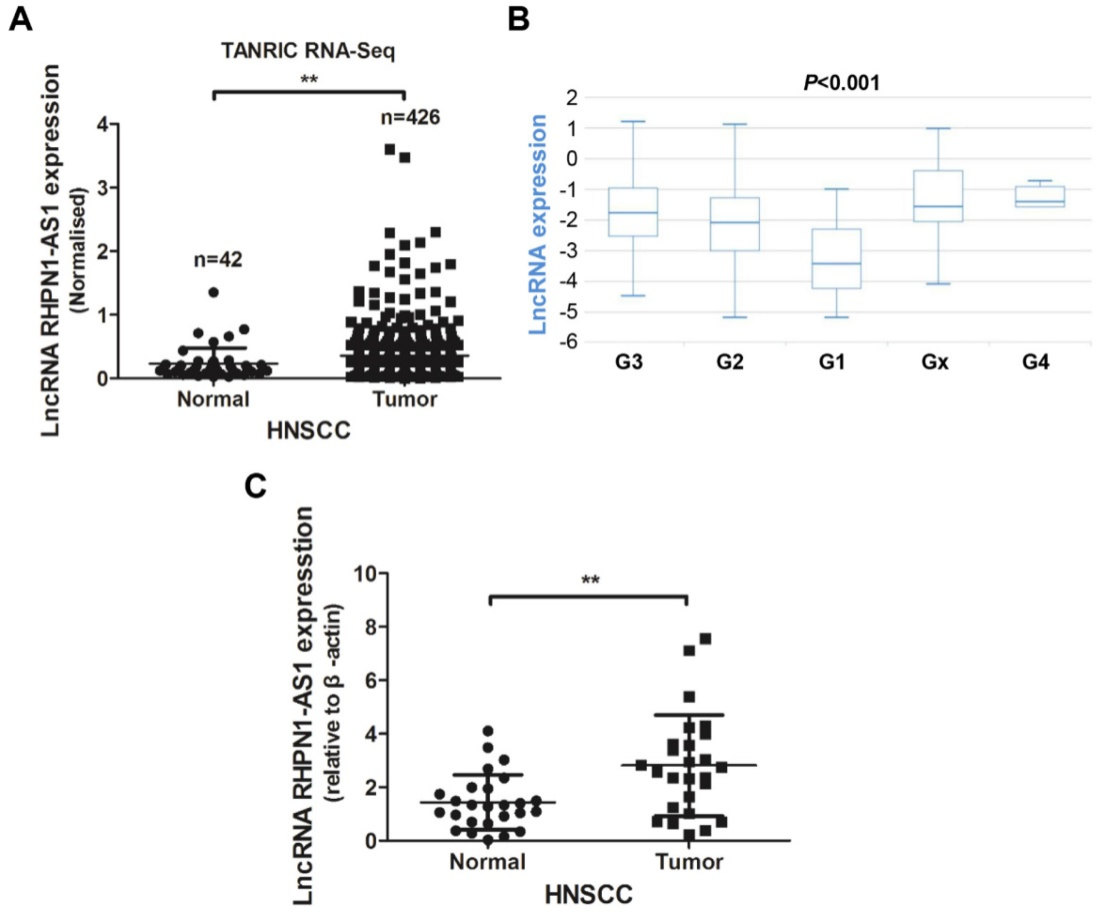
** LncRNA *RHPN1-AS1* was upregulated in HNSCC** (A) - (B) Data on *RHPN1-AS1* expression in HNSCC and normal tissues obtained from the TANRIC platform were analyzed, and *RHPN1-AS1* was upregulated in HNSCC tissues compared to normal tissues (426 tumor cases vs. 42 normal cases; *P* < 0.01), and *RHPN1-AS1* expression level was associated with neoplasm histologic grade (*P* < 0.001). (C) Relative expression of *RHPN1-AS1* in HNSCC tissues compared with that of adjacent normal tissues (n = 26, *P* < 0.01). *RHPN1-AS1* expression was determined using qPCR and was normalized to β-actin expression. The data represent the mean ± SD of 3 replicates. ** *P* < 0.01.

LncRNA *RHPN1-AS1* was recently identified as an oncoRNA in uveal melanoma (UM) 16. We analyzed the *RHPN1-AS1* expression levels in HNSCC tissues from the TANRIC platform. The analysis results revealed that *RHPN1-AS1* was upregulated in HNSCC tissues (426 tumor tissues vs. 42 normal tissues; Fig. 1A, *P* < 0.01), and the *RHPN1-AS1* level was correlated with neoplasm histologic grade (Fig. 1B, *P* < 0.001). In addition, patients with high *RHPN1-AS1* expression had poor survival compared to patients with low *RHPN1-AS1* expression; however, the *P* value was greater than 0.05 (*P* = 0.086, data not shown). To further verify the expression of *RHPN1-AS1*, we obtained 26 pairs of HNSCC and adjacent normal tissues (Table 1) to measure the lncRNA expression levels. QPCR results revealed that *RHPN1-AS1* was remarkably upregulated in HNSCC tissues (Fig. 1C, *P* < 0.01), which was in accord with our above results.

### Knockdown of *RHPN1-AS1* inhibited the migration and invasion of HNSCC cells

**Figure 2:**
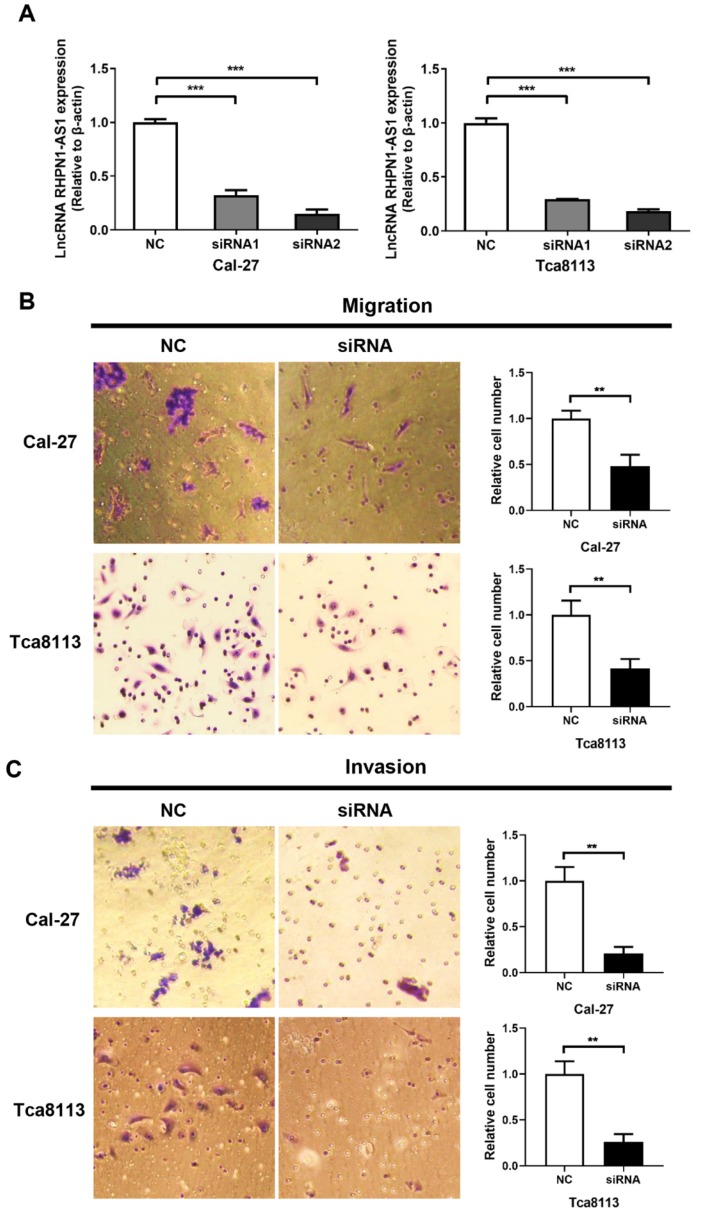

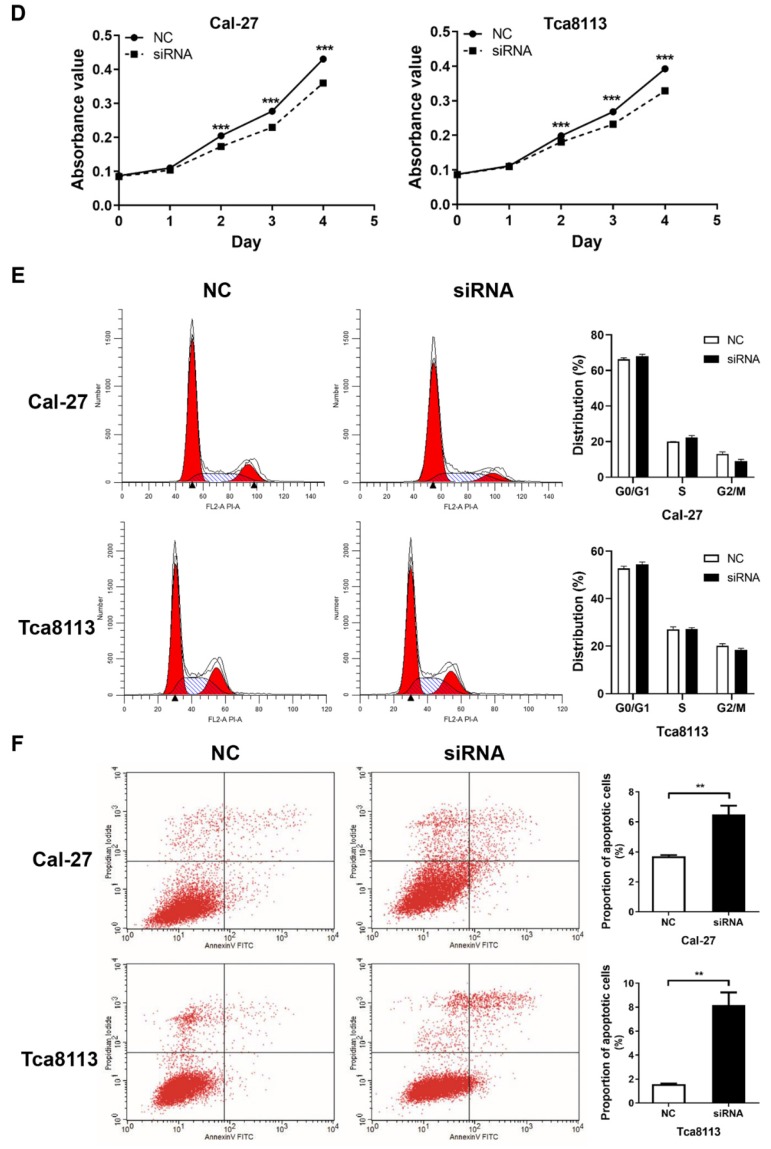
** RHPN1-AS1 knockdown inhibited HNSCC cell migration, invasion and proliferation (**A) Cal-27 and Tca8113 cells were transfected with RHPN1-AS1 siRNA1, RHPN1-AS1 siRNA2, and a negative control (NC). QPCR was used to detect the efficacy of the RHPN1-AS1 knockdown. (B) - (C) Cell migration and invasion were examined in Cal-27 and Tca8113 cells transfected with *RHPN1-AS1* siRNA or NC by Transwell assays without and with Matrigel coating in the upper compartment. (D) Measurement of growth curves of Cal-27 and Tca8113 cells transfected with *RHPN1-AS1* siRNA or NC by MTT assays. (E) - (F) Cell cycle distribution and apoptosis were determined by flow cytometry. The data represent the mean ± SD of 3 replicates. ** *P* < 0.01; ***P < 0.001.

To investigate whether *RHPN1-AS1* affected the malignant behavior of HNSCC cells, we transfected siRNAs targeting *RHPN1-AS1* (si-*RHPN1-AS1*) and negative control siRNA (NC) into Cal-27 and Tca8113 cells. Transfection efficiency was evaluated by qPCR (Fig. 2A, *P* < 0.001), and the second siRNA had the strongest effect. Therefore, we chose this siRNA in our subsequent experiments. Transwell assays were used to investigate cell migration and invasion. As shown in Fig. 2B, *RHPN1-AS1* knockdown significantly inhibited migration by approximately 50% in Cal-27 cells and by nearly 60% in Tca8113 cells (*P* < 0.01). As expected, a significant inhibition of invasion was observed after transfecting *RHPN1-AS1* siRNA into Cal-27 and Tca8113 cells compared with that of the NC group (Fig. 2C, *P* < 0.01).

### Knockdown of *RHPN1-AS1* blocked cell proliferation and promoted cell apoptosis in HNSCC

An MTT assay was performed to evaluate cell viability. The si-*RHPN1-AS1* group of both Cal-27 and Tca8113 cells showed slow proliferation compared with the control group (Fig. 2D, *P* < 0.001). Flow cytometric analysis was performed to detect the cell cycle and apoptosis. Transfection of *RHPN1-AS1*-siRNA did not affect the cell cycle distribution of Cal-27 and Tca8113 cells (Fig. 2E). However, the percentage of apoptotic cells was significantly increased after silencing *RHPN1-AS1* in both Cal-27 and Tca8113 cells (Fig. 2F, *P* < 0.01).

### *RHPN1-AS1* regulated the expression of epithelial-mesenchymal transition (EMT)-related genes

**Figure 3:**
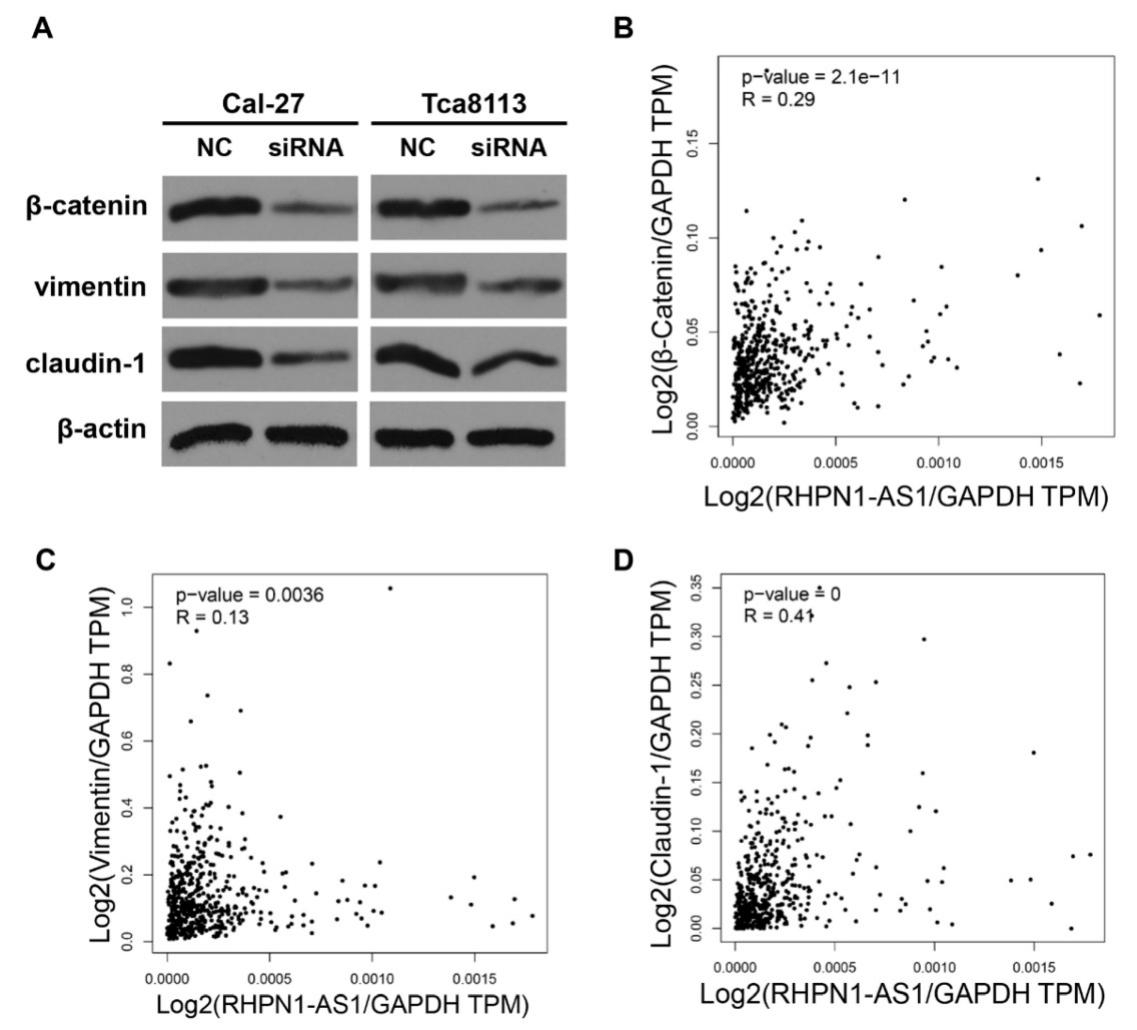
** RHPN1-AS1 knockdown suppressed the expression of EMT-related genes** (A) The protein levels of β-catenin, vimentin and claudin-1 were detected in Cal-27 and Tca8113 cells transfected with *RHPN1-AS1* siRNA or NC by western blotting. The expression of β-actin was detected as a protein loading control. (B)-(D) The correlation analysis between *RHPN1-AS1* and *β-Catenin*, *Claudin-1* and *Vimentin* expression levels in HNSCC was performed by using the GEPIA database.

EMT is known to facilitate tumor migration and invasion 17. In a previous study, a gene expression profile analysis revealed that *RHPN1-AS1* may participate in the EMT process through regulation of TGF-β signaling in UM 16. To illuminate the functional mechanisms of* RHPN1-AS1*, we detected the expression levels of several EMT-related genes by western blot analyses. The results showed that *RHPN1-AS1* knockdown significantly downregulated the expression of β-catenin, claudin-1 and vimentin in Cal-27 and Tca8113 cells (Fig. 3A). In addition, the correlation analysis between *RHPN1-AS1* and the *β-Catenin*, *Claudin-1* and *Vimentin* expression levels in HNSCC was performed by using the GEPIA database. The results indicated that *RHPN1-AS1* expression is positively correlated with *β-Catenin*, *Claudin-1* and *Vimentin* expression (Fig. 3B-D).

## Discussion

LncRNAs originate from different regions of the genome, including the promoters or untranslated regions of protein-coding genes, the antisense strand or the introns of protein-coding genes, or even independent transcripts within and outside of protein-coding genes 18, 19. These RNAs were previously considered as genetic byproducts and did not attract much attention in the past few decades because of the absence of biological functions 20, 21. However, recent reports have demonstrated that the majority of the human genome is transcribed into lncRNAs, and increasing evidence suggests that lncRNAs play a critical role in the regulation of diverse pathological and physiological processes 22. Currently, an increasing number of studies have identified “oncogene” or “tumor suppressor” lncRNAs in cancer 23, 24. To date, the dysregulated expression and involvement of lncRNAs have been reported in diverse cancers, including HNSCC 11, 24-26. For example, high expression of the lncRNA *KCNQ1OT1* was closely correlated with poor prognosis in tongue squamous cell carcinoma (TSCC) and promoted TSCC cell proliferation 27. Upregulation of *HOTAIR* promoted HNSCC cell invasion and metastasis and was associated with poor prognosis of patients 28, 29. *UCA1* was not only correlated with the lymph node metastasis of TSCC but could also enhance the migration of TSCC cells 30. In addition, Cao et al. 31 reported that *KTN1-AS1*, *LINC00460* and *RP5-894A10.6* act as novel biomarkers for the accurate prognostic prediction of patients with HNSCC by analyzing RNA-seq data derived from the TANRIC database. Therefore, lncRNAs have promising applications in new diagnostic and therapeutic strategies in HNSCC.

*RHPN1-AS1*, a 2030-bp transcript originating from human chromosome 8q24.3, is an antisense lncRNA derived from the promoter region of *RHPN1*. A previous study reported that *RHPN1-AS1* was upregulated in the UM and that knockdown of *RHPN1-AS1* significantly inhibited UM cell proliferation, migration and invasion 16. In another study, the authors developed a lncRNA network-based prioritization approach, named “LncNetP”, and predicted that *RHPN1-AS1* may serve as a novel disease risk factor for hepatocellular carcinoma diagnosis and prognosis 32. We first found that this lncRNA has abnormal expression levels in HNSCC based on large-scale samples by analyzing RNA-Seq data, strongly implying that *RHPN1-AS1* may function as a tumor promoter in HNSCC. Thus, fresh HNSCC tissues were collected, and cell lines were used for further investigation. As expected, *RHPN1-AS1* was significantly overexpressed in HNSCC tissues compared with adjacent normal tissues. Inhibition of *RHPN1-AS1* in Cal-27 and Tca8113 cells suppressed migration, invasion and cell viability, which is consistent with the findings of a previous study of UM 16. In addition, knockdown of *RHPN1-AS1* promoted HNSCC cell apoptosis, but it had no effect on the cell cycle. Collectively, our results suggest that *RHPN1-AS1*, acting as an oncogene, may be a potential diagnostic and therapeutic target in HNSCC.

Cell migration and invasion are significant aspects of cancer progression, and EMT plays an important role in tumor cell migration and invasion 33. EMT represents a transformation program underpinning the invasive and metastatic phenotype of cancers, which could be interpreted as a state of de-differentiation 34. This process can be induced by multiple signaling pathways, including the Wnt/β-catenin signaling pathway 35-37. Accumulating evidence indicates that many important oncogenes or tumor suppressors regulate the EMT program and thus are implicated in tumor cell migration and invasion 38. In addition, lncRNAs also participate in this regulatory process 34. The lncRNA* NKILA* inhibited migration and invasion of TSCC cells by suppressing EMT 39. *MALAT1* promoted TSCC cell growth and metastasis by regulating the activity of the Wnt/β-catenin signaling pathway 40, 41. In the present study, we tested the effect of *RHPN1-AS1* knockdown on EMT-related genes. Compared with the control group, the *RHPN1-AS1* knockdown group showed decreased protein expression of β-catenin, claudin-1 and vimentin in Cal-27 and Tca8113 cells, which are critical for EMT. In addition, we performed the correlation analysis between *RHPN1-AS1* and the *β-Catenin*, *Claudin-1* and *Vimentin* expression levels in HNSCC by using the GEPIA database. The results indicated that *RHPN1-AS1* expression is positively correlated with the expression of *β-Catenin*, *Claudin-1* and *Vimentin* in HNSCC. The above data indicated that *RHPN1-AS1* could regulate the expression of EMT-related genes in HNSCC cells.

Studies have revealed that lncRNAs function by direct regulation or indirect interaction with other molecules, such as protein, DNA, and RNA 22, 42-44. For example, lncRNAs may exert their functions through interactions with regulatory proteins, such as chromatin remodeling proteins 45. The lncRNA *HOTAIR* has been shown to interact with Polycomb Repressive Complex 2 components such as Ezh2 to regulate gene expression 46. The lncRNA *GClnc1* could be used as a molecular scaffold to recruit WDR5 and KAT2A complexes for specific modifications of histones 47. Additionally, lncRNAs can interact with RNA molecules and thus regulate gene expression as competing endogenous RNAs (ceRNAs), as described previously 22, 48. For instance, the lncRNA *SNHG15* acts as a ceRNA to regulate the YAP1-Hippo signaling pathway by sponging miR-200a-3p in papillary thyroid carcinoma 49. *H19* functions as a ceRNA to sponge the miRNA let-7 family, leading to an increase in the expression of let-7 targets in several cancers 48. In this study, our preliminary exploration suggests that *RHPN1-AS1* promotes tumor cell migration and invasion by regulating the expression of EMT-related genes in HNSCC cells. However, the mechanisms underlying how *RHPN1-AS1* regulates the expression of EMT-related genes remain unclear and should be further studied.

Every study, including the present study, has its limitations. First and foremost, there were no reciprocal experiments to examine the effects of *RHPN1-AS1* overexpression on the malignant phenotype of HNSCC cells. Additionally, we confirmed that *RHPN1-AS1* promotes tumor cell migration and invasion by regulating the expression of EMT-related genes in HNSCC cells. However, the mechanisms underlying *RHPN1-AS1-*mediated regulation of EMT-related gene expression remain unclear.

## Conclusion

In conclusion, this study demonstrates that lncRNA *RHPN1-AS1* was upregulated in HNSCC and that its expression level was correlated with neoplasm histologic grade. Knockdown of *RHPN1-AS1* inhibited the migration, invasion and proliferation of HNSCC cells. *RHPN1-AS1* promoted tumor development by regulating the expression of EMT-related genes. Collectively, our results suggest that *RHPN1-AS1*, acting as an oncogene, may be a potential diagnostic and therapeutic target in HNSCC.

## Figures and Tables

**Table 1:** The clinicopathological information of 26 HNSCC patients

No.	Gender	Age (years)	Diagnosis	Clinical stage	Histological grade
1	M	53	TSCC	IV (T2N2M0)	Moderate
2	M	63	TSCC	II (T2N0M0)	Moderate
3	M	55	BSCC	II (T2N0M0)	Moderate
4	M	65	TSCC	III (T3N1M0)	Well/Moderate
5	M	61	TSCC	III (T2N1M0)	Well/Moderate
6	M	66	BSCC	IV (T3N2M0)	Moderate
7	F	53	TSCC	III (T2N1M0)	Moderate /Poor
8	M	52	TSCC	II (T2N0M0)	Moderate
9	M	47	TSCC	II (T2N0M0)	Well/Moderate
10	M	68	TSCC	II (T2N0M0)	Well/Moderate
11	M	46	TSCC	I (T1N0M0)	Well
12	M	44	TSCC	I (T1N0M0)	Well
13	F	38	BSCC	II (T2N0M0)	Well/Moderate
14	M	52	BSCC	II (T2N0M0)	Well
15	M	37	TSCC	IV (T2N2M0)	Poor
16	M	50	TSCC	II (T2N0M0)	Well
17	M	55	TSCC	I (T1N0M0)	Moderate/Poor
18	M	62	TSCC	IV (T2N2M0)	Well/Moderate
19	M	54	TSCC	III (T3N0M0)	Well/Moderate
20	M	57	TSCC	II (T2N0M0)	Well
21	M	53	TSCC	IV (T3N2M0)	Well/Moderate
22	M	36	TSCC	II (T2N0M0)	Moderate
23	M	45	TSCC	IV (T2N2M0)	Well/Moderate
24	M	51	BSCC	III (T3N0M0)	Well
25	M	46	TSCC	I (T1N0M0)	Well
26	M	44	TSCC	I (T1N0M0)	Well

Abbreviations: M, male; F, female; TSCC, tongue squamous cell carcinoma; BSCC, buccal squamous cell carcinoma.
